# Ethyl Acetate Extracts of Semen Impatientis Inhibit Proliferation and Induce Apoptosis of Human Prostate Cancer Cell Lines through AKT/ERK Pathways

**DOI:** 10.1155/2017/1243515

**Published:** 2017-03-13

**Authors:** Tao Wang, Yang Cai, Wen Song, Ruibao Chen, Dunmei Hu, Jianhan Ye, Lu Liu, Wei Peng, Junfeng Zhang, Shaogang Wang, Weiming Yang, Jihong Liu, Yufeng Ding

**Affiliations:** ^1^Department of Urology, Tongji Hospital, Tongji Medical College, Huazhong University of Science and Technology, Wuhan 430030, China; ^2^Institute of Urology, Tongji Hospital, Tongji Medical College, Huazhong University of Science and Technology, Wuhan 430030, China; ^3^Department of Pharmacy, Tongji Hospital, Tongji Medical College, Huazhong University of Science and Technology, Wuhan 430030, China

## Abstract

*Objective.* To investigate the inhibitory effect of ethyl acetate extracts of* Impatiens balsamina* L. on prostate cancer cells.* Methods. Impatiens balsamina* L. was extracted to get water, ethanol, oil ether, ethyl acetate, and butanol extracts. CCK-8 assay was used to detect the inhibitory effect. Apoptosis rates and cell cycle distribution were detected by flow cytometry. Transwell assay was performed to test the ability of migration. The expressions of Bcl-2, Bax, cleaved-caspase-3, p-ERK, ERK, p-AKT, AKT, cyclin D1, cyclin E, and MMP2 were detected by Western blot.* Results.* Ethyl acetate extracts had the strongest inhibitory effect. After being treated with different concentrations of ethyl acetate extracts, the percentage of G0/G1 phase increased significantly, cyclin D1 and cyclin E expression decreased, apoptosis rate was significantly higher, and the ability of migration of PC-3 and RV1 was inhibited significantly. Western blot showed that the expressions of Bcl-2, p-ERK, and p-AKT were significantly decreased, but the expressions of Bax and caspase-3 cleavage were increased.* Conclusions. Impatiens balsamina* L. inhibited the proliferation of human prostate cancer cells; ethyl acetate extracts have the strongest effect. It could inhibit cell proliferation and migration, cause G1 phase arrest, and induce apoptosis probably through inhibition of the AKT and ERK pathways.

## 1. Introduction

Prostate cancer is the most frequent prevalent cancers and the second highest cause of mortality by cancer for the male population in western countries [1] and has a relatively low incidence in Asia. But the incidence and mortality data in Asia had a remarkable increase in the past 30 years [2]. For early and localized PCa, surgery, hormone ablation, and radiation therapy are the most commonly utilized treatments. For advanced and metastasized tumors, chemotherapy is the only choice, but it has limited efficacy and could lead to various side effects in patients. So, it is very necessary and urgent to develop a new agent for the treatment of prostate cancer. Recently, growing evidences suggest that many herbal medicines possess anticancer activity and are considered as an important alternative to the conventional treatments for cancers [3].

Semen Impatientis is the dried ripe seeds of* Impatiens balsamina* L. which has been largely cultivated in China for medicinal purpose. It has been officially recorded in Chinese Pharmacopoeia as a traditional Chinese herbal medicine for the treatment of amenorrhea, abdominal mass, bone choking throat, and sores [4]. Extensive research over the past decades has shown that the bioactive compounds isolated from* Impatiens balsamina* L. have antimicrobial, antioxidant, and anticancer activities [5, 6]. Previous reports revealed that* Impatiens balsamina* L. induced growth inhibition and apoptosis in human oral squamous cell carcinoma cell lines [7]. 2-Methoxy-1,4-naphthoquinone from* Impatiens balsamina* L. exhibited high ability to induce gastric adenocarcinoma apoptosis through the caspase-dependent apoptosis pathway [8]. Ding et al. have shown that ethanol or chloroform extracts of* Impatiens balsamina* L. have antitumor activity against the human hepatocellular carcinoma cell line HepG2 [6]. However, the antitumor effects of Semen Impatientis on prostate cancer have not yet been investigated. The potential molecular mechanism underlying the effects needs to be explored as well.

This study was undertaken to investigate whether the extracts of Semen Impatientis affect cell growth and to clarify its potential molecular mechanism in human prostate cancer cells. The results of the present study demonstrated that Semen Impatientis inhibited the growth and metastatic of prostate cancer cells and induced apoptosis as well as G0/G1 phase cell cycle arrest. And the inhibitory effect of Semen Impatientis against prostate cancer cells was associated with decreased level of phosphorylated AKT and ERK. This may offer a promising new approach in the effective treatment of prostate cancer.

## 2. Materials and Methods

### 2.1. Cell Lines and Cell Culture

PC-3, RV1, and LNCaP cell lines were purchased from Shanghai Institute of Cell Biology (Shanghai, China). Cells were maintained in Roswell Park Memorial Institute medium 1640 (HyClone, USA) for PC-3 and LNCaP and Dulbecco's modified Eagle's medium (HyClone, USA) for RV1, supplemented with 10% fetal bovine serum (GIBCO, USA) and penicillin/streptomycin (100 U/mL and 100 mg/mL, resp.). All cell lines were maintained in a humidified incubator at 5% CO_2_ and 37°C.

### 2.2. Preparation of Different Extracts of Semen Impatientis

Semen Impatientis were purchased from Yi Yuan Chinese herbal medicine company. Dried, pulverized powder of Semen Impatientis (1000 g) was macerated in 95% ethanol for 24 h. After that, the ethanol extract was filtered. After extraction, the residue was distilled to get water extracts, Mixed water extracts, and ethanol extracts and then extracted by petroleum ether, ethyl acetate, and butanol separately to get petroleum ether extracts, ethyl acetate extracts, and butanol extracts.

### 2.3. Cell Proliferation Assay

Cell viability was assessed by Cell Counting Kit-8 (Dojindo, Japan) assay according to the manufacturer's instructions. Cells were reseeded in 96-well plates at 1.0 × 10^3^ cells per well for a final volume of 100 *μ*l. After incubation for adherence, the cells were treated with all five extracts of Semen Impatientis at different concentrations for 24 hours. Medium was then removed and 100 *μ*l of PBS containing 10 *μ*l Cell Counting Kit-8 was added to each well. After 2 hours of incubation at 37°C, the absorbance at 450 nm was measured using a multiwell microplate reader (PerkinElmer, Waltham, MA). Each experiment was performed in triplicate and the data represent the mean of all measurements.

### 2.4. Cell Cycle Analysis

5 × 10^5^ cells per well were seeded in a 6-well plate and incubated overnight. Cells were treated with different concentrations of EAESI (0, 40, and 80 *μ*g/ml) for 24 h. Then cells were harvested with trypsinization and fixed with 70% ice-cold ethanol at 4°C overnight. After ethanol treatment, the cells were washed with ice-cold PBS. After incubation with 100 mg/ml RNase A (Sigma, USA) and 50 mg/ml propidium iodide (PI) for 30 min at 37°C in the dark, cells were finally analyzed by flow cytometry (Becton Dickinson, USA).

### 2.5. Apoptosis Assay

5 × 10^5^ cells per well were seeded in a 6-well plate and incubated overnight. Cells were treated with different concentrations of EAESI (0, 40, and 80 *μ*g/ml) for 24 h. Then cells were harvested, washed with ice-cold PBS, resuspended in 100 *μ*l binding buffer containing 5 *μ*l of FITC-conjugated Annexin V and 5 *μ*l of PI (50 *μ*g/mL), and incubated for another 30 minutes in the dark. Cells were finally analyzed by flow cytometry (Becton Dickinson, USA).

### 2.6. Migration Assay

Cell migration was detected by the Transwell assay. After being treated with EAESI for 6 h, 1 × 10^5^ cells were seeded in upper chamber with serum-free medium containing 0.3% BSA and medium containing 10% fetal bovine serum was added to the lower chamber. After further incubation for 48 hours, cells that had not migrated were discarded from the upper chamber of the Transwell using a cotton swab. Cells that had migrated to the bottom of the chamber were fixed in methanol and stained with 1% of crystal violet. The migrated cells of random five fields of each chamber were counted under a microscope at 100x magnification field.

### 2.7. Western Blot Analysis

The prostate carcinoma cells were treated with different concentrations of EAESI (0, 40, and 80 *μ*g/ml). After 24 h of incubation, cells were collected and lysed with cell lysis buffer. Protein concentration was detected by the BCA protein assay kit. Equal amounts of total protein (30 *μ*g) were separated by 10% sodium dodecyl sulphate-polyacrylamide gel electrophoresis (SDS-PAGE), transferred onto polyvinylidene fluoride membranes (Millipore, USA), blocked with 5% nonfat milk in tris-buffered saline for 2 h at room temperature, and then incubated with the specific primary antibody solution overnight at 4°C. After incubation with HRP-linked secondary antibody for 1 h at 37°C, bands were visualized by incubation with ECL (Millipore, USA). The experiments were repeated three times.

### 2.8. Statistical Analysis

All results were expressed as mean ± standard deviation (SD) for three independent experiments. The statistical significance of differences between means was calculated using One-way Analysis of Variance (ANOVA) by SPSS 19.0. The value of *P* < 0.05 was considered statistically significant.

## 3. Results

### 3.1. Inhibitory Effects of Semen Impatientis Extracts on the Proliferation of Human Prostate Cancer Cells

**Figure 1:**
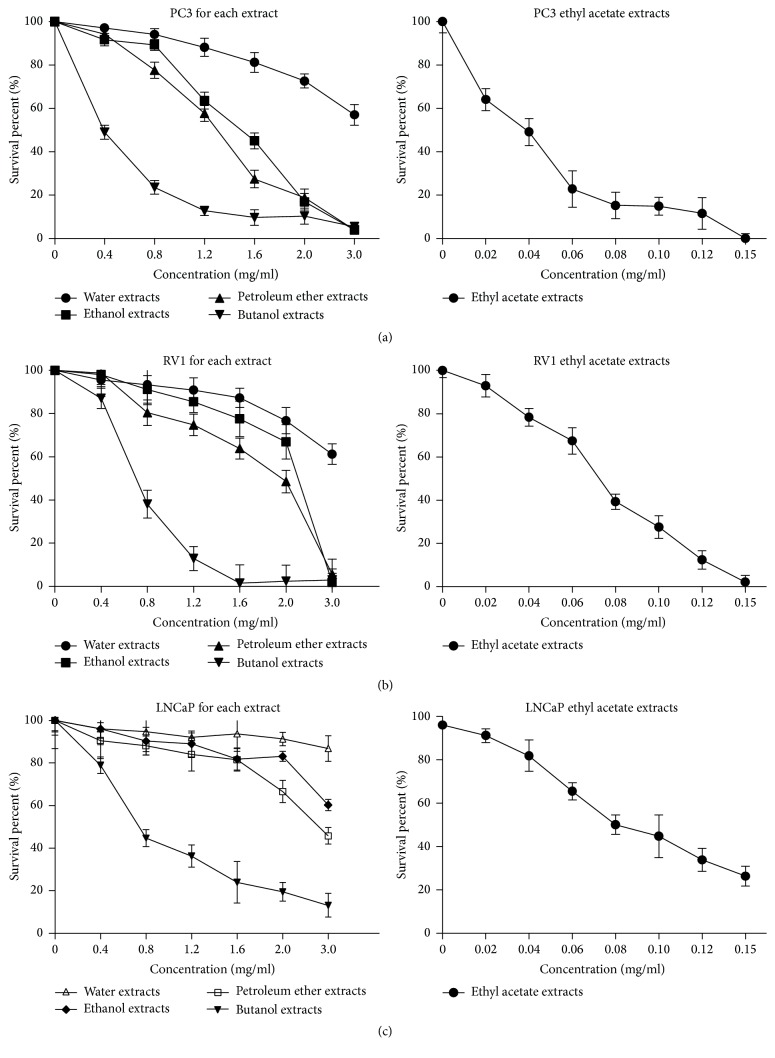
Extracts of* Impatiens balsamina* L. inhibit the proliferation of human prostate cancer cells. ((a)–(c)) PC3, RV1, and LNCaP cells were treated with various concentrations of each of the extracts of* Impatiens balsamina* L. for 24 h. The cell viability was determined using a CCK8 assay. All data are expressed as mean ± SE.

CCK-8 assay was applied to evaluate the antiproliferation effect of Semen Impatientis extracts on human prostate cancer cells in vitro. The growth curves of human prostate cancer cells following various concentrations of Semen Impatientis extracts for 24 h were shown in Figure 1. The results showed that Semen Impatientis extracts had different degrees of antiproliferative activity on human prostate cancer cells, while ethyl acetate extracts of Semen Impatientis (EAESI) had the strongest inhibitory effect on the growth of PC-3, RV1, and LNCaP cells (IC50 = 32, 69 and 85 *μ*g/ml, respectively), compared to the other extracts of Semen Impatientis. (Figure 1) And EAESI significantly inhibited the growth of PC-3, RV1, and LNCaP cells in a dose-dependent manner (*P* < 0.05). Based on these observations, we selected a dose of 40–80 *μ*g/ml of EAESI treatment for further mechanism studies.

### 3.2. Effects of EAESI on the Induction of Apoptosis in Human Prostate Cancer Cells

**Figure 2:**
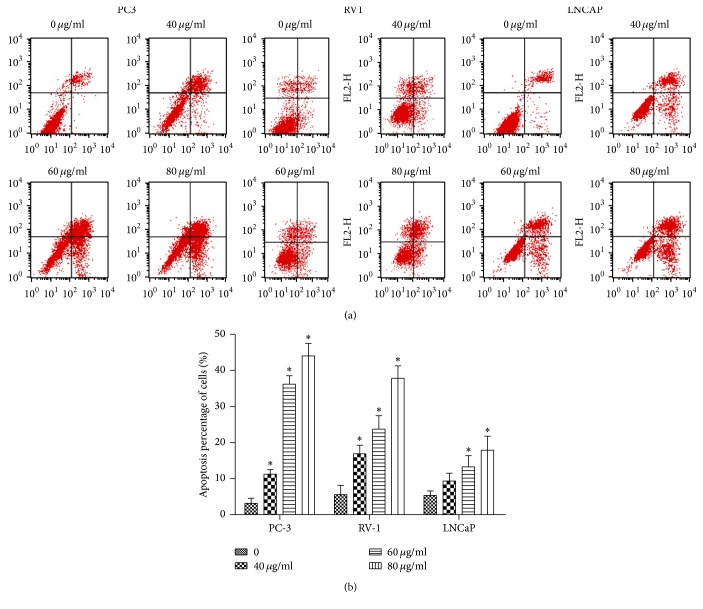
EAESI induces apoptosis in PC3, RV1, and LNCaP cells. PC3, RV1, and LNCaP cells were treated with different concentrations (0, 40, and 80 *μ*g/ml) of EAEIB for 24 h. (a) The apoptosis effect was determined by Annexin V-FITC/PI staining. (b) The data are expressed as mean ± SD (*n* = 3), with results representative of 3 independent experiments shown. ^*∗*^*P* < 0.05 versus the control group.

Apoptosis induced by EAESI was evaluated using Annexin V-FITC/PI staining assay. Flow cytometric analysis showed that, after incubation with 40, 60, and and 80 *μ*g/ml EAESI for 24 h, the apoptosis rate of PC-3 cells was 11.23 ± 1.29%, 36.17 ± 2.35%, and 44.00 ± 3.48%, which were significantly higher than that of the control group (3.15 ± 1.42%, *P* < 0.05, Figure 2). Similar results were obtained in RV1 cells and LNCaP cells. The data indicated that EAESI induced the apoptosis of PC-3, RV1, and LNCaP cells in a dose-independent manner.

### 3.3. Effects of EAESI on the Induction of G0/G1 Phase Arrest in Human Prostate Cancer Cells

**Figure 3:**
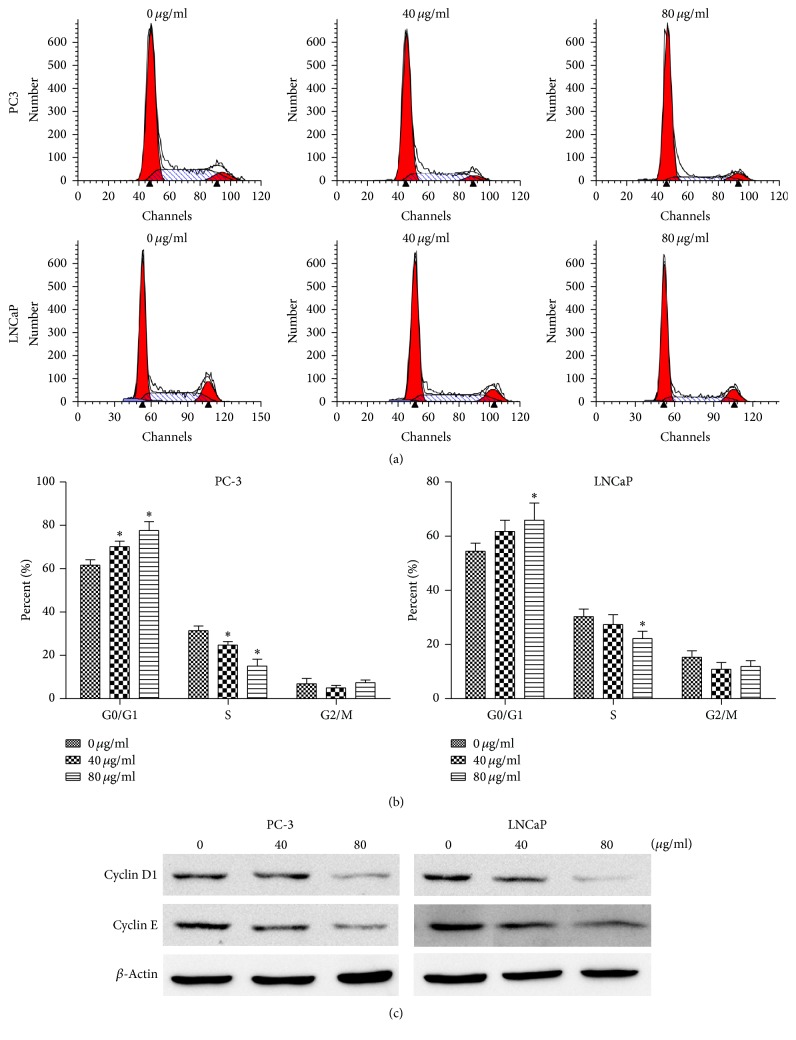
EAESI induces cell cycle arrest at G0/G1 phase in PC3 and LNCaP cells. PC3 and LNCaP cells were treated with different concentrations (0, 40, and 80 *μ*g/ml) of EAEIB for 24 h. (a) The cell cycle distribution was determined by PI staining assay. (b) The data are expressed as mean ± SD (*n* = 3), with results representative of 3 independent experiments shown. ^*∗*^*P* < 0.05, versus the control group. (c) The expression of cyclin D1 and cyclin E proteins was determined by Western blot analysis.

To determine whether EAESI induced the arrest of cell cycle progression in PC-3 and LNCaP cells, PI staining assay was applied to quantitate the cell cycle distribution after treatment with different concentrations of EAESI (0, 40, and 80 *μ*g/ml). After incubation with EAESI 24 h, the percentage of G0/G1 phase population in PC3 cell was noticeably enhanced by 70.2% and 77.6%, which were significantly higher than the control group 61.7% (*P* < 0.05, Figure 3). In addition, the percentage of G0/G1 phase population in LNCaP cell was noticeably enhanced by 61.8% and 65.9%, which were significantly higher than the control group 54.4%. Meanwhile, the percentage of S and G2/M phases cells decreased after treatment. Cyclin D1 and cyclin E are important regulators of G1-S phase cell cycle transition. The expression levels of cyclin D1 and cyclin E were determined by Western blotting. The results showed that, after the treatment of EAESI, cyclin D1 and cyclin E expression in PC3 and LNCaP decreased. The results indicated that EAESI could induce cell cycle arrest at the G0/G1 phase in PC-3 and LNCaP cells.

### 3.4. Inhibitory Effects of EAESI on the Migration of Human Prostate Cancer Cells

**Figure 4:**
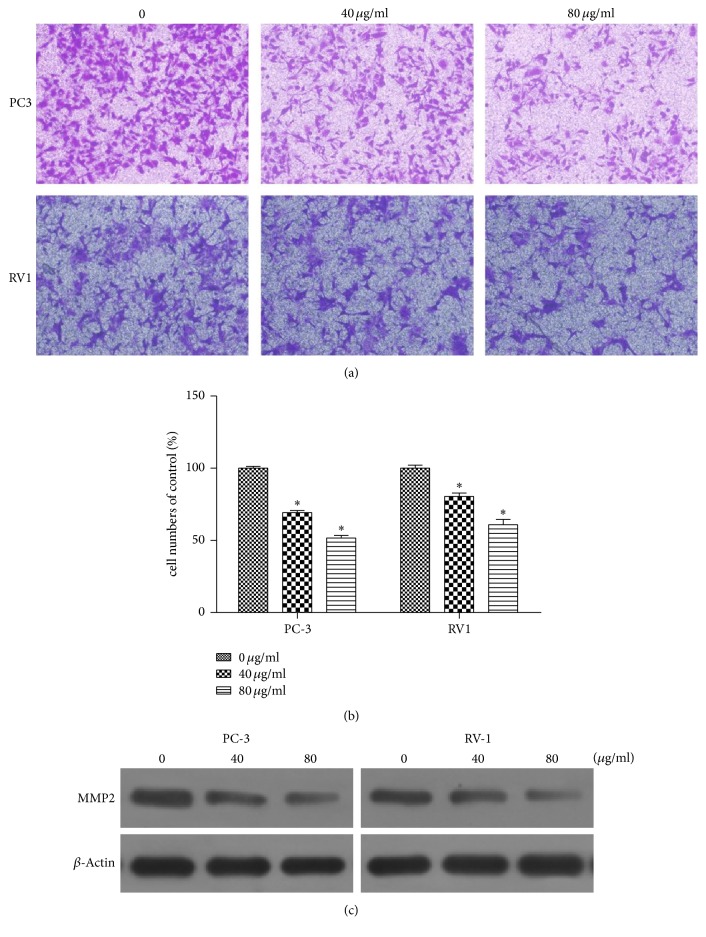
EAESI inhibit migration in PC3 and RV1 cells. PC3 and RV1 cells were treated with different concentrations (0, 40, and 80 *μ*g/ml) of EAEIB for 48 h. Analysis of change in migration on PC3 and RV1 cells by Transwell assay. (a) The cells penetrated the insert membrane were stained with crystal violet and photographed under a light microscope at ×100. (b) Quantitation of the number of cells that penetrated the insert membrane. The data are expressed as mean ± SD (*n* = 3), with results representative of 3 independent experiments shown. ^*∗*^*P* < 0.05 versus the control group. (c) The expression level of MMP2 was determined by Western blot. *β*-Actin was used as a loading control.

The migration potential of the cells was determined by Transwell Matrigel migration assay system. As shown in Figure 4, after incubation with 40 and 80 *μ*g/ml EAESI 48 h, the PC-3 cell migration ability decreased to 69.4 ± 1.29% and 51.9 ± 1.48% compared to the control and the RV1 cell migration ability decreased to 80.50 ± 2.35% and 61.10 ± 3.48% compared to the control (*P* < 0.05, Figure 4). There was a significant reduction in cellular migration of PC-3 and RV1 cells at 80 *μ*g/ml EAESI. Treatment of PC3 cells with EAESI resulted in a more pronounced inhibition of migration compared to RV1 cells. MMP-2 was closely associated with cell migration and invasion. To further investigate the effect of EAESI on the migration of human prostate cancer cells, Western blot was performed to detect changes in the expression levels of MMP-2 after incubation with 40 and 80 *μ*g/ml EAESI. Figure 4 revealed that the protein expression levels of MMP-2 were decreased. The results indicated that EAESI could inhibit cell migration in PC3 and RV1 cells.

### 3.5. Effects of EAESI on the Expression of Apoptosis-Related Proteins

**Figure 5:**
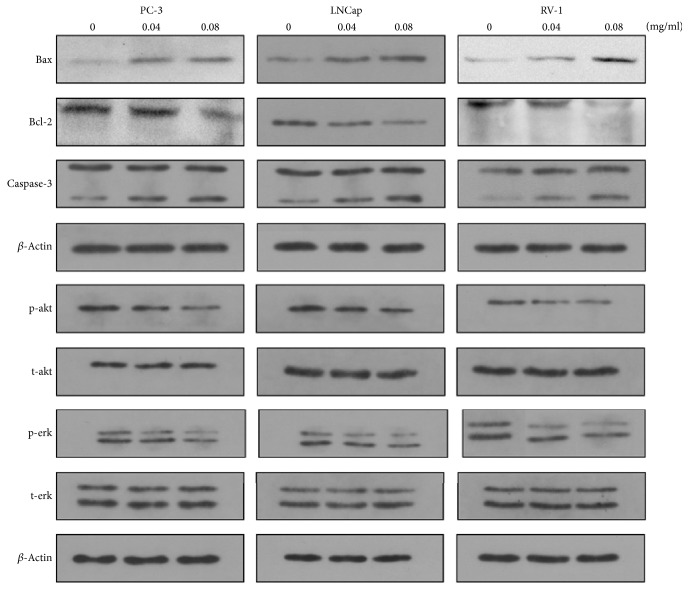
EAESI induces apoptosis in PC3, RV1, and LNCaP cells through AKT ERK pathway. PC3, RV1, and LNCaP cells were treated with different concentrations (0, 40, and 80 *μ*g/ml) of EAEIB for 24 h. The expression levels of BAX, Bcl-2, caspase-3, Akt, phospho-Akt, ERK, and phospho-ERK were determined by Western blot. *β*-Actin was used as a loading control. The results are representative of 3 independent experiments.

To investigate the mechanism of apoptosis, cells were treated with 0, 40, and 80 *μ*g/ml EAESI for 24 hours and then the expressions of apoptosis-related proteins such as Bcl-2, Bax, and caspase-3 were detected by Western blot. After treatment with various concentration of EAESI, the expression levels of Bcl-2 were significantly decreased, but the expressions of Bax and caspase-3 cleavage were decreased compared with the control group (Figure 5). These results indicated that the EAESI induced apoptosis in PC-3, RV1, and LNCaP cells via activation of caspase-3 which resulted from increase of Bax and decrease of Bcl-2.

### 3.6. Effects of EAESI on Raf/MEK/ERK and PI3K/AKT Signaling

Akt and ERK play a critical role in cell proliferation, differentiation, and survival. To evaluate the role of EAESI on AKT pathway and ERK pathway, cells were treated with various concentrations of EAESI (0, 40, and 80 *μ*g/ml) for 24 h; then the levels of Akt, phospho-Akt, ERK, and phospho-ERK in whole cell lysates were determined by Western blot. As shown in Figure 5, treatment with EAESI significantly decreased phospho-AKT and phospho-ERK levels in a dose-independent manner but had no effect on the total levels of AKT and ERK. These data indicated that the induction of apoptosis by EAESI maybe is mediated by AKT pathway and ERK pathway.

## 4. Discussion

Semen Impatientis is the dried ripe seeds of* Impatiens balsamina* L., as a traditional Chinese herbal medicine, mainly used for treatment of amenorrhea, abdominal mass, bone choking throat, and sores [4]. In recent years, several studies have shown that Semen Impatientis can inhibit the growth and induce apoptosis of a variety of tumor cells. For instance, Shin et al. found that Semen Impatientis could inhibit growth and induce apoptosis in human oral squamous cell carcinoma cell lines [7]. Wang and Lin found that Semen Impatientis could induce gastric adenocarcinoma apoptosis through the caspase-dependent apoptosis pathway [8]. Ding et al. have shown that* Impatiens balsamina* have antitumor activity against the human hepatocellular carcinoma cell line HepG2 [6]. However, the effects of Semen Impatientis on prostate cancer have not yet been investigated. In the present study, we found that Semen Impatientis could inhibit the growth and induce apoptosis of prostate cancer in vivo.

We chose androgen-dependent prostate cancer cell LNCaP and androgen-independent prostate cancer cells RV1 and PC-3 for this study. Semen Impatientis was extracted and separated to get water extracts, ethanol extracts, petroleum ether extracts, ethyl acetate extracts, and butanol extracts. Cell Counting Kit-8 (CCK-8) assay was used to detect the inhibitory effect of different extract of Semen Impatientis on human prostate cancer cell lines LNCaP, RV1, and PC-3. In our current study, the extracts of Semen Impatientis showed a dose-dependent inhibitory effect in growth of PC-3, RV1, and LNCaP cells and ethyl acetate extracts have the strongest effect (IC50 = 32, 69 and 85 *μ*g/ml, resp.). So we selected a dose of 40–80 *μ*g/ml of ethyl acetate extracts for subsequent research.

Cancer metastasis is the leading cause of mortality in patients with prostate cancer. Thus, preventing prostate cancer metastasis is the key target to improve prognosis. The results showed that EAESI 40–80 *μ*g/ml remarkably inhibited PC-3 and cells LNCaP cells migration, a highly metastatic human prostate carcinoma cell line. It indicated that EAESI could inhibit prostate cancer cells metastasis.

Abnormal apoptosis and cell cycle regulation are two important reasons for infinite proliferation of malignant tumor. Deregulation of cell cycle progression is a universal characteristic of majority of cancer cell growth. The present flow cytometric analysis in PC3 cells demonstrated that the number of cells in G0/G1 was significantly increased and cells in G2/M were decreased after the treatment of EAESI. Similar significant rise in G0/G1 cells was also found in LNCaP cells after the treatment with EAESI. Cyclin D1 and cyclin E are critical for the G1-S transition of the cell cycle and induce G1 arrest when its level is too low. Therefore, a decrease in cyclin D1 levels may be suggested as one of the reasons for the G0/G1 arrest of the PC3 and LNCaP cell cycle, which resulted in the inhibition of cell growth in the presence of EAESI. This suggested that EAESI caused G0/G1 cell cycle arrest in human prostate cancer cells.

Apoptosis is a physiological process of programmed cell death. Suppression of apoptosis is a recognized hallmark of cancer [9], and causing cell apoptosis plays major roles in many conventional anticancer therapies [10]. Several studies have shown that there are kinds of natural products having the ability to induce apoptosis in various human tumor cells [11]. Therefore, we examined whether the inhibitory effects of EAESI toward prostate cancer cells were attributed to apoptosis. The results of Annexin V-FITC/PI staining demonstrated that EAESI treatment could induce prostate cancer cells apoptosis in a dose-dependent manner. Apoptosis pathway is regulated by a balance of proapoptotic and antiapoptotic members of the Bcl-2 family of proteins, which include Bax, Bcl-2, and Bid proteins. Bax is traditionally known as a proapoptotic protein [12] which could induce changes in the mitochondrial potential upon apoptotic stimuli and lead to cytochrome c releasing, while Bcl-2 is regarded as apoptosis inhibitors in the cells. Our data showed that EAESI decreased the expression of prosurvival proteins Bcl-2 and increased the expression of the proapoptotic molecule Bax in a dose-dependent manner. Caspase-3, as one of the key executioners of apoptosis, can systematically dismantle cells by cleaving many key proteins. Our results showed that the expression of cleaved-caspase-3 was significantly increased. Thus our results suggest that EAESI induced apoptosis might belong to caspase-dependent manner.

PI3K/AKT and Raf/MEK/ERK play a prominent role in the promotion of growth and the prevention of apoptosis [13]. AKT, the central mediator of the PI3K/AKT pathway, promotes cell survival by inhibiting proapoptotic Bcl-2 family members BAD and BAX. Increased levels of Akt are detected in prostate cancer and are associated with poorer prognosis. Some prostate cancer cell lines such as LNCaP and PC3 cells express high levels of active Akt [14]. Increased expression of the Raf/MEK/ERK pathway has been associated with advanced prostate cancer, hormonal independence, and poor prognosis [15]. Elevated ERK protect cells from apoptosis by phosphorylating and inhibiting caspase-9 [16]. Inhibition of the Raf/MEK/ERK and PI3K/Akt pathways is usually an effective method to induce apoptosis. Some reports have revealed that AKT inactivation could mediate cell cycle arrest and apoptosis. Cho et al. found that methanol extracts of Semen Impatientis induced growth inhibition and apoptosis in OSC-20, human OSCC cells, which were mediated by decreasing Akt expression and dephosphorylating p-Akt [17]. Li et al. found that formononetin could inhibit cell proliferation and induce cell apoptosis as well as G1 cell cycle arrest by inactivation of Akt [18]. In our study, we found that EAESI suppressed the phosphorylation of AKT and ERK in LNCaP and PC-3 cells. Those results indicated that EAESI induced apoptosis in prostate cancer cells by AKT and ERK inactivation.

In conclusion, our results demonstrated that EAESI could inhibit cell proliferation, inhibit cell migration, cause G1 phase arrest, and induce apoptosis probably through inhibition of the AKT and ERK pathways in human prostate cancer cells. These results suggest that EAESI may be a potential candidate for prostate cancer treatment.
